# Identification and validation of energy metabolism-related genes in diabetic kidney disease through integrated bioinformatics and in vivo analysis

**DOI:** 10.1186/s41065-026-00632-7

**Published:** 2026-01-28

**Authors:** Hui Jiang, Ming-Hui Geng, Yue-Mei Zhan, Jin-Feng Shen, Fu-Zhen Wang, Sen-Qing Lin, Zhe Hong, Chun-Hua Guo, Jin-Xiu Deng, Sen-Chao Wu

**Affiliations:** Department of Nephrology, Longyan First Affiliated Hospital of Fujian Medical University, No.105 Jiuyi North Road, Xinluo District, Longyan, Fujian Province 364000 China

**Keywords:** Bioinformatics analysis, Diabetic kidney disease, Energy metabolism, Reactive oxygen species

## Abstract

**Background:**

The primary renal complication of diabetes mellitus is diabetic kidney disease (DKD). The precise pathogenic mechanisms of DKD remain poorly elucidated. The aim of this study was to identify potential energy metabolism-related genes associated with DKD.

**Methods:**

The GSE30529 and GSE30528 datasets were retrieved from the Gene Expression Omnibus, and energy metabolism-related genes were obtained from the GeneCards database. Differentially expressed genes (DEGs) between DKD and control groups were analyzed. The biological functions and signaling pathways of these DEGs were evaluated using Gene Ontology (GO), the Kyoto Encyclopedia of Genes and Genomes (KEGG), and gene set enrichment analysis (GSEA). The diagnostic performance of hub genes for DKD was assessed using receiver operating characteristic (ROC) curve analysis. Expression levels of five significant energy metabolism-related genes were validated through immunohistochemistry. The Nephroseq V5 tool was used to evaluate gene expression in DKD and to determine correlations between gene expression and renal function in patients with DKD.

**Results:**

A total of 17 energy metabolism-related DEGs were identified. Five hub genes—*ALB*, *IGF1*, *CD36*, *LPL*, and *UCP2*—were identified. Among these, *CD36* and *LPL* demonstrated relatively high diagnostic accuracy for DKD. The findings suggest that *CD36*, *IGF1*, *LPL*, and *UCP2* may serve as potential biomarkers for DKD.

**Conclusions:**

The genes *CD36*,* IGF1*,* LPL*, and *UCP2* represent potential energy metabolism-related biomarkers with possible applications in the diagnosis and treatment of DKD.

**Supplementary Information:**

The online version contains supplementary material available at 10.1186/s41065-026-00632-7.

## Background

Alterations in the renal metabolic network contribute to the onset and progression of diabetic kidney disease (DKD). Patients with diabetes who do not have kidney disease exhibit an approximately 30-fold lower risk of all-cause mortality compared to those with DKD. The presence of DKD markedly increases morbidity among patients with diabetes mellitus, underscoring the need for a more comprehensive understanding of its etiology to guide the development of effective therapeutic strategies [1–3]. The early stages of DKD are often clinically asymptomatic, and by the time albuminuria is detected, pathological lesions have typically advanced, accelerating renal decline toward end-stage renal disease. Early diagnosis and treatment of DKD can halt or significantly delay progression [4]. However, the underlying pathogenic mechanisms remain poorly understood, requiring further investigation [5]. 

Previous research has examined the pathophysiological processes of DKD from multiple perspectives. Monitoring alterations in energy metabolism has emerged as a potential method for identifying specific and sensitive biomarkers, and therapeutic targets, relevant to DKD onset and progression. Evidence indicates that early DKD is associated with disruptions in renal energy metabolism, including glucose metabolite accumulation and disturbances in the tricarboxylic acid (TCA) cycle, ultimately resulting in mitochondrial dysfunction in advanced stages of the disease. As an organ with high energy demand, the kidney is largely dependent on mitochondrial function [6]. However, multiple studies have revealed that DKD is often accompanied by varying degrees of mitochondrial dysfunction and structural abnormalities, and these phenomena may be closely related to hyperglycemia and lipid metabolism disorders [7]. Under physiological conditions, intracellular free fatty acids, which serve as a key energy source, are efficiently metabolized in mitochondria mainly through the β-oxidation pathway. However, the hyperglycemic environment and insulin resistance in diabetes disrupt the balance between fatty acid synthesis and oxidation, resulting in lipid metabolism disorders [8]. These findings will provide new directions for the diagnosis and treatment of DKD. Metabolomic analyses have identified several uremic toxins, such as tryptophan derivatives and phenyl sulfate, as potential biomarkers of DKD progression [3]. Studies have shown that a variety of natural active ingredients (such as flavonoids, terpenoids and polyphenols) can effectively break the vicious circle of lipid peroxidation by regulating the balance of fatty acid synthesis and oxidation, enhancing the antioxidant defense system and inhibiting inflammatory signal transduction, thereby exhibiting broad application prospects in the field of DKD treatment [9]. Despite these findings, further in-depth investigation into DKD-associated energy metabolism is warranted.

Advances in bioinformatics and microarray technologies have enabled the detection of DNA mutations at the genomic level. In this context, the present study aimed to identify hub genes related to energy metabolism in DKD through an integrated analysis of publicly available datasets using bioinformatics methods. The findings may contribute to improved strategies for the prevention and treatment of DKD.

## Materials and methods

### Data acquisition

Two DKD-related datasets, GSE30529 and GSE30528, were obtained from the Gene Expression Omnibus (GEO) database using the R package GEOquery [10, 11]. The R package sva was applied to remove batch effects between GSE30529 and GSE30528 [12]. The combined datasets were standardized, probe annotations were performed, and normalization was conducted using the R package limma [13]. Detailed dataset information is presented in Table 1.

**Table 1:** GEO microarray chip information

	GSE30529	GSE30528
Platform	GPL571	GPL571
Species	Homo sapiens	Homo sapiens
Tissue	Kidney	Kidney
Samples in DN group	10	9
Samples in Control group	12	13
Reference	PMID: 21,752,957	PMID: 21,752,957

*GEO* Gene Expression Omnibus,* DN* Diabetic Nephropathy

Energy metabolism-related genes (EMRGs) were retrieved from the GeneCards database, restricted to “Protein Coding” genes with a relevance score > 3 and using the keyword “energy metabolism.” [14] The “Relevance score” in GeneCards represents the degree of association between a gene and the keyword (energy metabolism); setting “Relevance score > 3” aims to filter protein-coding genes with moderate to high relevance to the biological function of energy metabolism, thereby improving the specificity and reliability of the resulting gene set, an additional 22 EMRGs reported in published literature were obtained from PubMed using the same keyword [14]. After merging and deduplication, a total of 252 EMRGs were identified. Detailed information is presented in Table S1.

### Differential analysis

The R package limma was used to identify differentially expressed genes (DEGs) between DKD and control groups, applying thresholds of |logFC| > 0.5 and adjusted *p* < 0.05. A volcano plot was generated using the R package ggplot2. To identify energy metabolism-related differentially expressed genes (EMRDEGs), DEGs were intersected with EMRGs. Venn diagrams (Wayne plots) were used to visualize the intersection. Heatmaps of EMRDEGs were generated with the R package *pheatmap*, and chromosomal locations were mapped using the R package *RCircos* [15]. 

### Functional enrichment analysis

Gene Ontology (GO) and Kyoto Encyclopedia of Genes and Genomes (KEGG) pathway enrichment analyses of EMRDEGs were performed using the R package clusterProfiler [16–18]. Statistically significant was defined as adjusted *p* < 0.05, with false discovery rate (FDR) controlled at < 0.25, using the Benjamini–Hochberg (BH) method for *p* adjustment.

### Gene set enrichment analysis

The R package clusterProfiler was used to perform gene set enrichment analysis (GSEA) on all genes in the combined datasets [19]. Gene sets containing 10–500 genes were included, with a random seed of 2020. The C2 gene sets were obtained from the Molecular Signatures Database [20]. Statistical significance was defined as an adjusted *p* < 0.05, with FDR < 0.25, using the BH correction.

### Protein–protein interaction network and hub gene screening

The STRING database was used to construct a PPI network of EMRDEGs, applying a minimum interaction score of 0.40 (medium confidence). Five algorithms in the Cytoscape plug-in cytoHubba—Maximal Clique Centrality (MCC), Degree, Maximum Neighborhood Component (MNC), Edge Percolated Component (EPC), and Closeness—were applied to rank EMRDEGs [21–23]. MCC reflects the centrality of genes in closely connected subnetworks, suitable for identifying key nodes within core modules. Degree: Degree centrality algorithm, measures the number of direct connections of a node, used to evaluate local connectivity. MNC: Based on the size of the largest connected subgraph in the node’s neighborhood, reflects the degree of local network aggregation. EPC: Evaluates a node’s ability to propagate information through edge percolation in the network, reflecting local robustness. Closeness: Reflects the average distance between a node and other nodes in the network, representing global propagation efficiency.

The top 10 genes from each algorithm were identified, and the intersecting genes were defined as energy metabolism-related hub genes. A Venn diagram was used to visualize the overlaps. This intersection strategy can reduce the bias of a single algorithm and ensure the robustness of the screening results.

### Regulatory network

MicroRNAs (miRNAs) targeting hub genes were retrieved from the TarBase database [24]. The mRNA–miRNA regulatory network was constructed using Cytoscape. Transcription factors (TFs) regulating hub gene expression were obtained from the ChIPBase and hTFTarget databases [25, 26]. Only TFs with a combined “Number of samples found (upstream)” and “Number of samples found (downstream)” > 4 were included. This standard refers to the recommended parameters from ChIPBase v2.0 and the hTFtarget database to improve the reproducibility and reliability of regulatory relationships. The databases used (TarBase, ChIPBase, hTFtarget) all integrate high-confidence data based on experimental validation (such as ChIP-seq, CLIP-seq, etc.).

### Differential expression and receiver operating characteristic analysis of hub genes

Group comparison plots were generated for hub gene expression levels. Receiver operating characteristic (ROC) curves and the corresponding area under the curve (AUC) values were calculated using the R package pROC.

### Immune infiltration analysis

Single-sample gene set enrichment analysis (ssGSEA) was performed using the R package GSVA to estimate immune cell infiltration scores. Samples with *p* < 0.05 were included in the immune infiltration matrix. Heatmaps showing correlations between immune cells and hub genes, as well as among immune cell types, were created using the R package pheatmap.

### Immunohistochemical staining

Paraffin-embedded kidney tissue samples were obtained from 10 patients with a clinical and histopathological diagnosis of diabetic nephropathy. Six paracancerous renal tissue specimens from patients with normoglycemic renal malignancy served as normal controls. All specimens were provided by the Longyan First Affiliated Hospital of Fujian Medical University and were approved by the institutional ethics committee (Approval No: LYREC2024-k025-01). The study was conducted in accordance with the ethical principles of the Declaration of Helsinki. Written informed consent was obtained from each patient for sample collection, preservation, and data use. Tissue preparation involved sectioning paraffin-embedded samples into 5-µm slices, which were mounted on glass slides, baked at 60 °C for 1 h, and deparaffinized in xylene. Rehydration was performed sequentially with 99%, 95%, and 70% ethanol. Sections were then incubated overnight at 4 °C with the following primary antibodies: *ALB* (GB14005, Servicebio, 1:200), *CD36* (AB252922, Abcam, 1:1000), *IGF1* (GB11248, Servicebio, 1:1000), *LPL* (55208-1-AP, proteintech, 1:200), and *UCP2* (AB97931, Abcam, 1:200). After primary antibody incubation, sections were treated with appropriate secondary antibodies. Staining was visualized using 3,3′-diaminobenzidine tetrahydrochloride (CW0125M; CWBIO) following the manufacturer’s instructions. Microscopic examination and imaging were performed with a BX43 microscope (Olympus). Quantitative analysis was conducted using ImageJ software.

### Clinical co-rrelation analysis

The Nephroseq V5 platform was used to assess the expression of *ALB*, *CD36*, *IGF1*, *LPL*, and *UCP2* genes in renal disorders. Correlations between the expression levels of these genes and the glomerular filtration rate (GFR) were evaluated in patients with DKD.

### Statistical analysis

All statistical analyses were performed using R software version 4.3.0. Continuous variables are expressed as mean ± standard deviation. The Wilcoxon rank-sum test was applied for comparisons between two groups. Unless otherwise specified, Spearman’s correlation was applied to calculate correlation coefficients. A *p* < 0.05 was considered statistically significant.

## Results

### Analysis flowchart

**Fig. 1:**
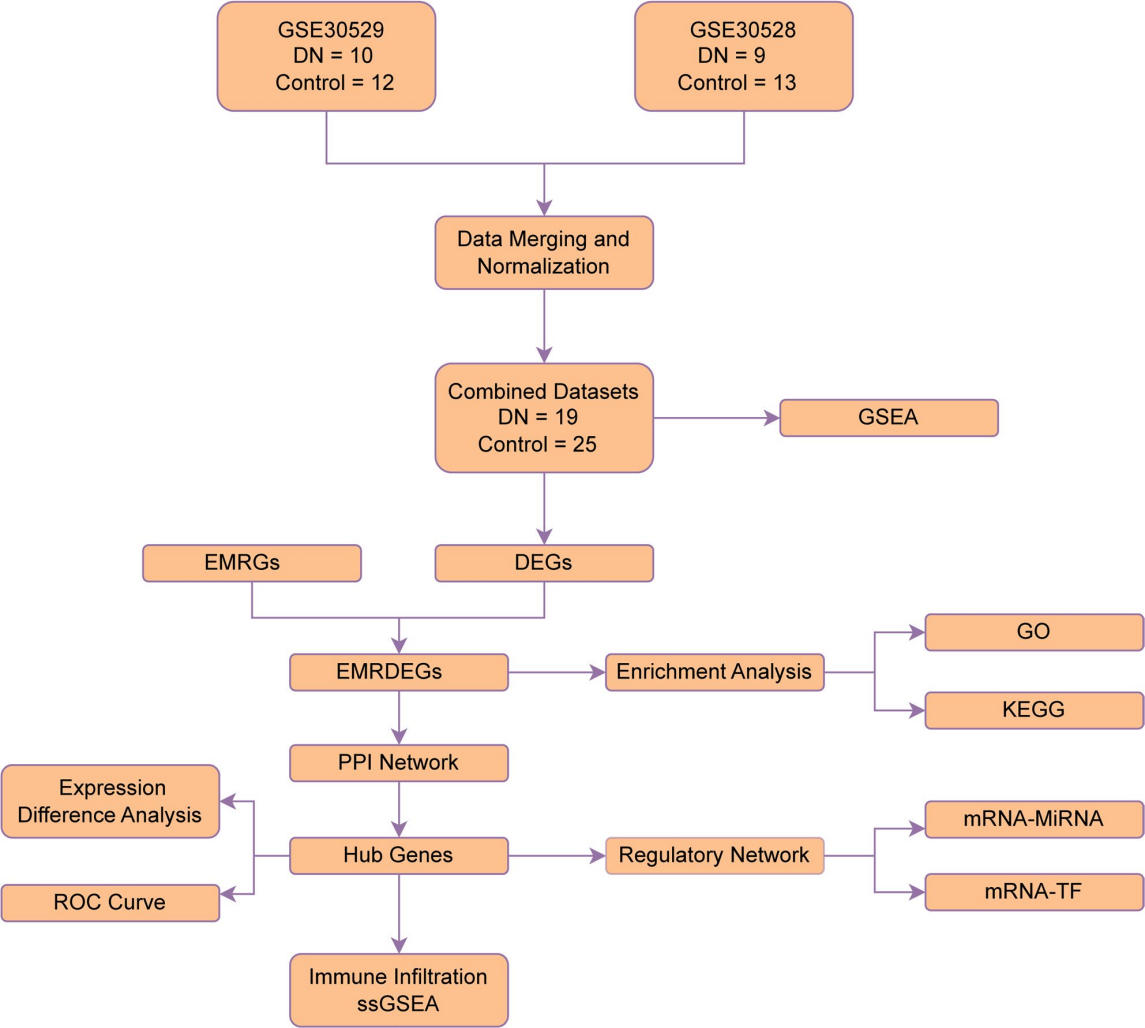
Flow chart for the comprehensive analysis of EMRDEGs. GEO, Gene Expression Omnibus; DKD, diabetic kidney disease; GO, gene ontology; KEGG, Kyoto Encyclopedia of Genes and Genomes; EMRDEGs, energy metabolism-related differentially expressed genes; DEGs, differentially expressed genes; ROC, receiver operating characteristic; GSEA, gene set enrichment analysis; EMRGs, energy metabolism-related genes; PPI, protein–protein interaction; ssGSEA, single-sample gene set enrichment analysis

The technical pathway for the bioinformatics analysis is presented in Fig. 1.

### Difference analysis of dataset-DKD

**Fig. 2:**
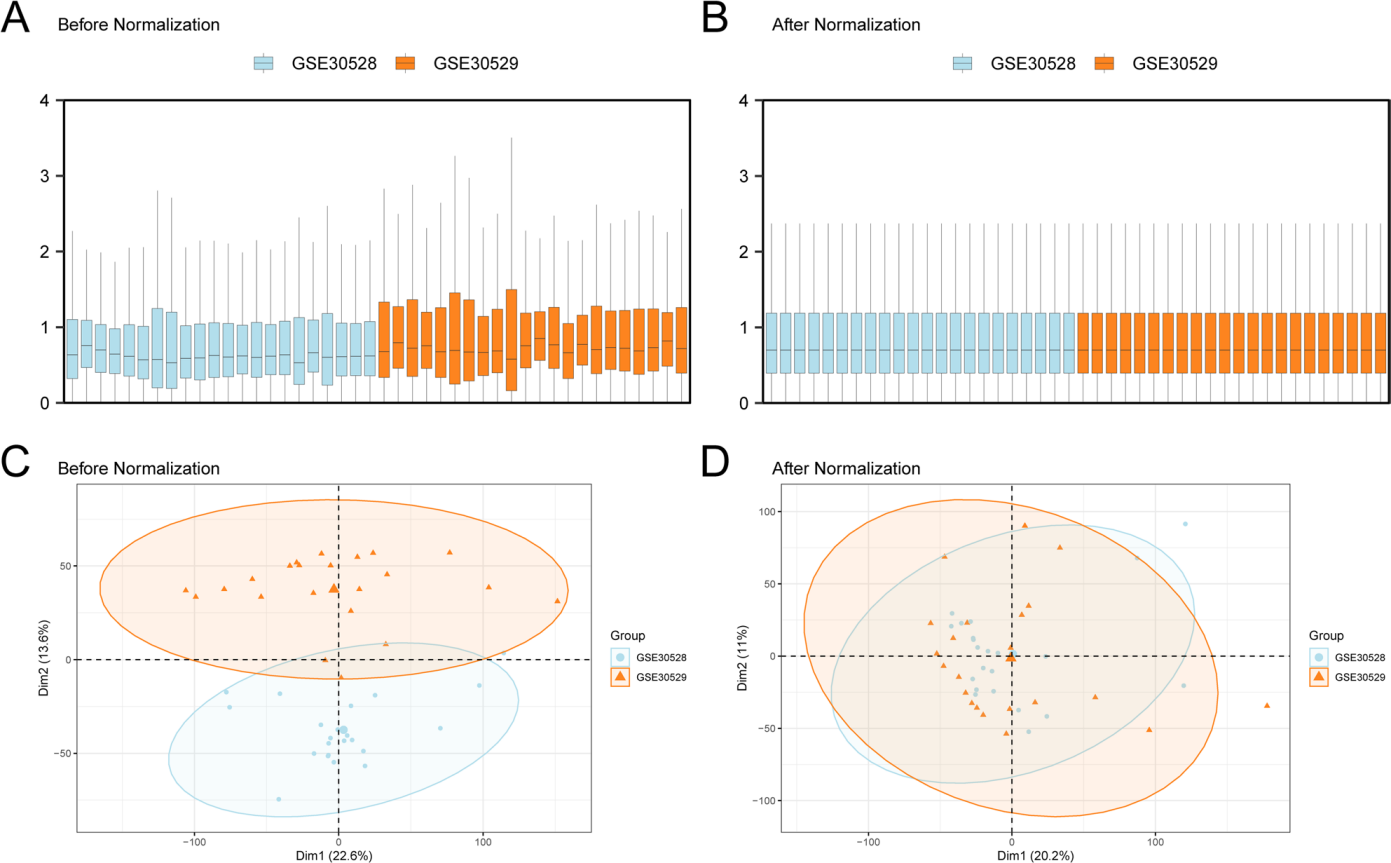
Batch effects removal of GSE30529 and GSE30528. **A** Box plot of dataset distribution before going to batch. **B** Go to the box plot of the combined GEO datasets distribution after batch processing. **C** PCA plot of the datasets before de-batching. **D** Go to the PCA map of the combined GEO datasets after batch processing. Orange and blue represent GSE30529 and GSE30528, respectively. PCA, principal component analysis

Distribution boxplots and principal component analysis (PCA) plots were used to compare the datasets before and after batch-effect removal (Fig. 2A–D). Following correction, the batch effect in the DKD dataset was reduced.

**Fig. 3:**
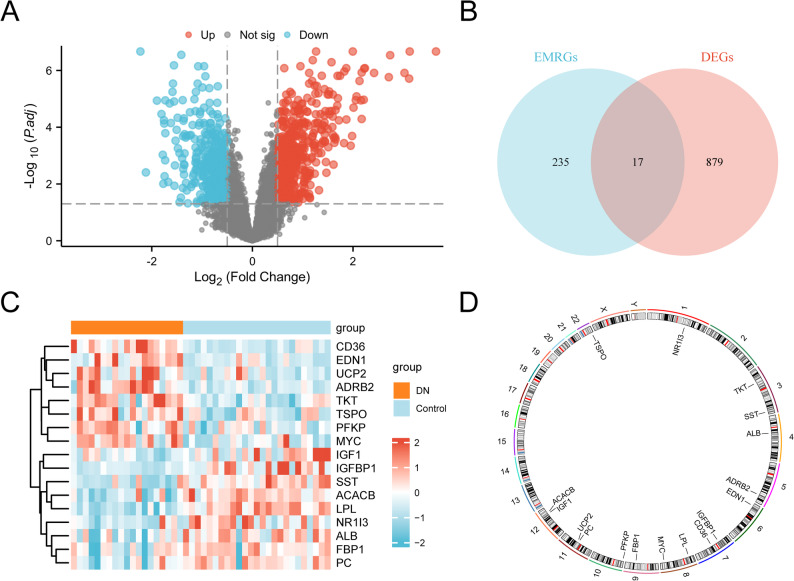
Differential gene expression analysis. **A**. Volcano plot of DEGs analysis between the DKD and control groups in combined GEO datasets. **B**. DEGs and EMRGs Venn diagram in the combined datasets. **C**. Heat map of expression values of EMRDEGs in the combined datasets. **D**. Chromosomal mapping of EMRDEGs. Orange and blue represent the DKD and control groups, respectively. GEO, Gene Expression Omnibus; DKD, diabetic kidney disease; EMRDEGs, energy metabolism-related differentially expressed genes; DEGs, differentially expressed genes; EMRGs, energy metabolism-related genes

In the combined datasets, 896 DEGs met the criteria of |logFC| > 0.5 and *p* < 0.05. Among these, 518 genes were upregulated (logFC > 0.5, *p* < 0.05), and 378 genes were downregulated (logFC < − 0.5, *p* < 0.05). A volcano plot was generated to visualize these findings (Fig. 3A). To identify EMRDEGs, the DEGs meeting the threshold criteria were intersected with EMRGs, and the overlap was visualized with a Venn diagram (Fig. 3B). A total of 17 EMRDEGs were identified: *UCP2*, *IGF1*, *TKT*, *CD36*, *ADRB2*, *TSPO*, *ACACB*, *LPL*, *FBP1*, *SST*, *IGFBP1*, *PFKP*, *NR1I3*, *MYC*, *ALB*, *PC*, and *EDN1*. Expression differences between sample groups in the combined GEO datasets were displayed in a heatmap generated using the R package pheatmap (Fig. 3C).

Chromosome mapping using the R package RCircos (Fig. 3D) revealed that EMRDEGs were predominantly distributed across chromosomes 3, 7, 8, 11, and 12. For example, *TKT* and *SST* were located on chromosome 3; *IGFBP1* and *CD36* on chromosome 7; and *LPL* and *MYC* on chromosome 8. *UCP2* and *PC* were mapped to chromosome 11, while *ACACB* and *IGF1* were located on chromosome 12.

### GO/KEGG

**Fig. 4:**
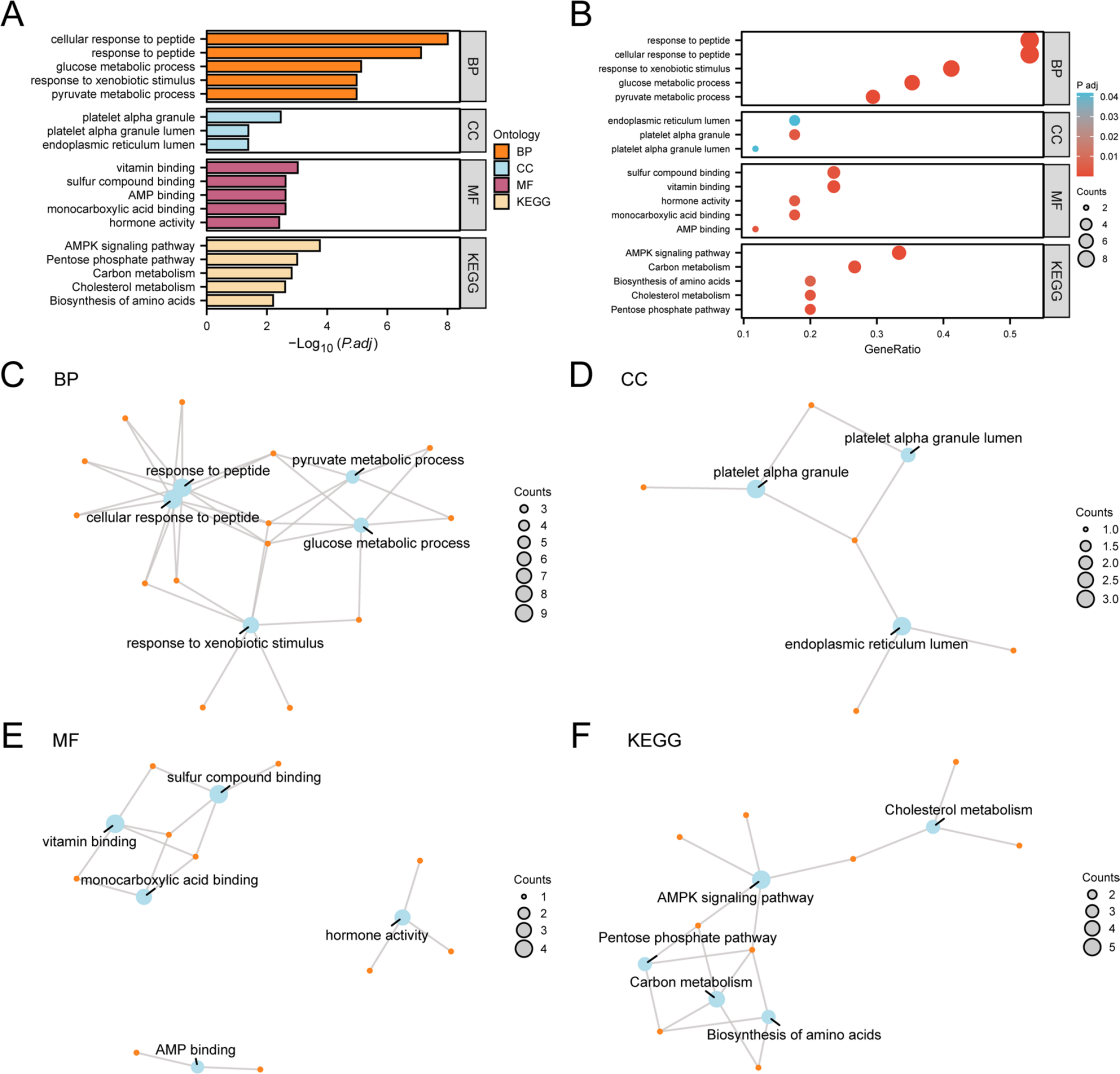
GO and KEGG enrichment analysis for EMRDEGs. **A**. Bar graph of GO and KEGG enrichment analysis results of EMRDEGs. **B**. Bubble diagram of GO and KEGG enrichment analysis results of EMRDEGs. **C–F**. GO and KEGG enrichment analysis results of EMRDEGs: BP (**C**), CC (**D**), MF (**E**), and KEGG (**F**). Objects are represented by blue nodes, molecules by orange nodes, and the relationship between objects and molecules is shown by lines. GO, gene ontology; KEGG, Kyoto Encyclopedia of Genes and Genomes; EMRDEGs, energy metabolism-related differentially expressed genes; BP, biological processes; CC, cell component; MF, molecular function

GO and KEGG enrichment analyses of the 17 EMRDEGs (Table 2) indicated significant enrichment in biological processes (BPs), including cellular response to peptide, glucose metabolic process, response to xenobiotic stimulus, and pyruvate metabolic process. Enriched cellular components (CCs) included platelet alpha granule, platelet alpha granule lumen, and endoplasmic reticulum lumen. Enriched molecular functions (MFs) included vitamin binding, sulfur compound binding, AMP binding, monocarboxylic acid binding, and hormone activity. KEGG pathway analysis demonstrated enrichment in the AMPK signaling pathway, pentose phosphate pathway, carbon metabolism, cholesterol metabolism, and amino acid biosynthesis. Results were visualized with bar (Fig. 4A) and bubble plots (Fig. 4B), as well as network diagrams for BP, CC, MF, and biological pathways (Figs. 4C–F).

**Table 2:** Result of GO and KEGG enrichment analysis for EMRDEGs

ONTOLOGY	ID	GeneRatio	BgRatio	pvalue	*p*.adjust	qvalue
BP	GO:1,901,653	9/17	361/18,800	6.82E-12	9.79E-09	4.15E-09
BP	GO:1,901,652	9/17	491/18,800	1.06E-10	7.61E-08	3.23E-08
BP	GO:0006006	6/17	201/18,800	1.56E-08	7.44E-06	3.15E-06
BP	GO:0006090	5/17	106/18,800	3.04E-08	1.05E-05	4.44E-06
BP	GO:0009410	7/17	411/18,800	3.65E-08	1.05E-05	4.44E-06
CC	GO:0031091	3/17	91/19,594	6.29E-05	3.46E-03	2.05E-03
CC	GO:0031093	2/17	67/19,594	1.52E-03	4.17E-02	2.47E-02
CC	GO:0005788	3/17	311/19,594	2.28E-03	4.19E-02	2.48E-02
MF	GO:0019842	4/17	148/18,410	8.80E-06	9.50E-04	3.89E-04
MF	GO:0033293	3/17	81/18,410	5.34E-05	2.40E-03	9.81E-04
MF	GO:0016208	2/17	15/18,410	8.37E-05	2.40E-03	9.81E-04
MF	GO:1,901,681	4/17	267/18,410	8.87E-05	2.40E-03	9.81E-04
MF	GO:0005179	3/17	122/18,410	1.80E-04	3.90E-03	1.60E-03
KEGG	hsa04152	5/15	121/8164	1.76E-06	1.76E-04	1.37E-04
KEGG	hsa00030	3/15	30/8164	1.98E-05	9.89E-04	7.70E-04
KEGG	hsa01200	4/15	115/8164	4.52E-05	1.51E-03	1.17E-03
KEGG	hsa04979	3/15	51/8164	9.91E-05	2.48E-03	1.93E-03
KEGG	hsa01230	3/15	75/8164	3.13E-04	6.26E-03	4.88E-03

*GO* Gene Ontology,* BP* Biological Process,* CC* Cellular Component,* MF* Molecular Function, *KEGG* Kyoto Encyclopedia of Genes and Genomes,* EMRDEGs* Energy Metabolism-Related Differentially Expressed Genes

### GSEA

**Fig. 5:**
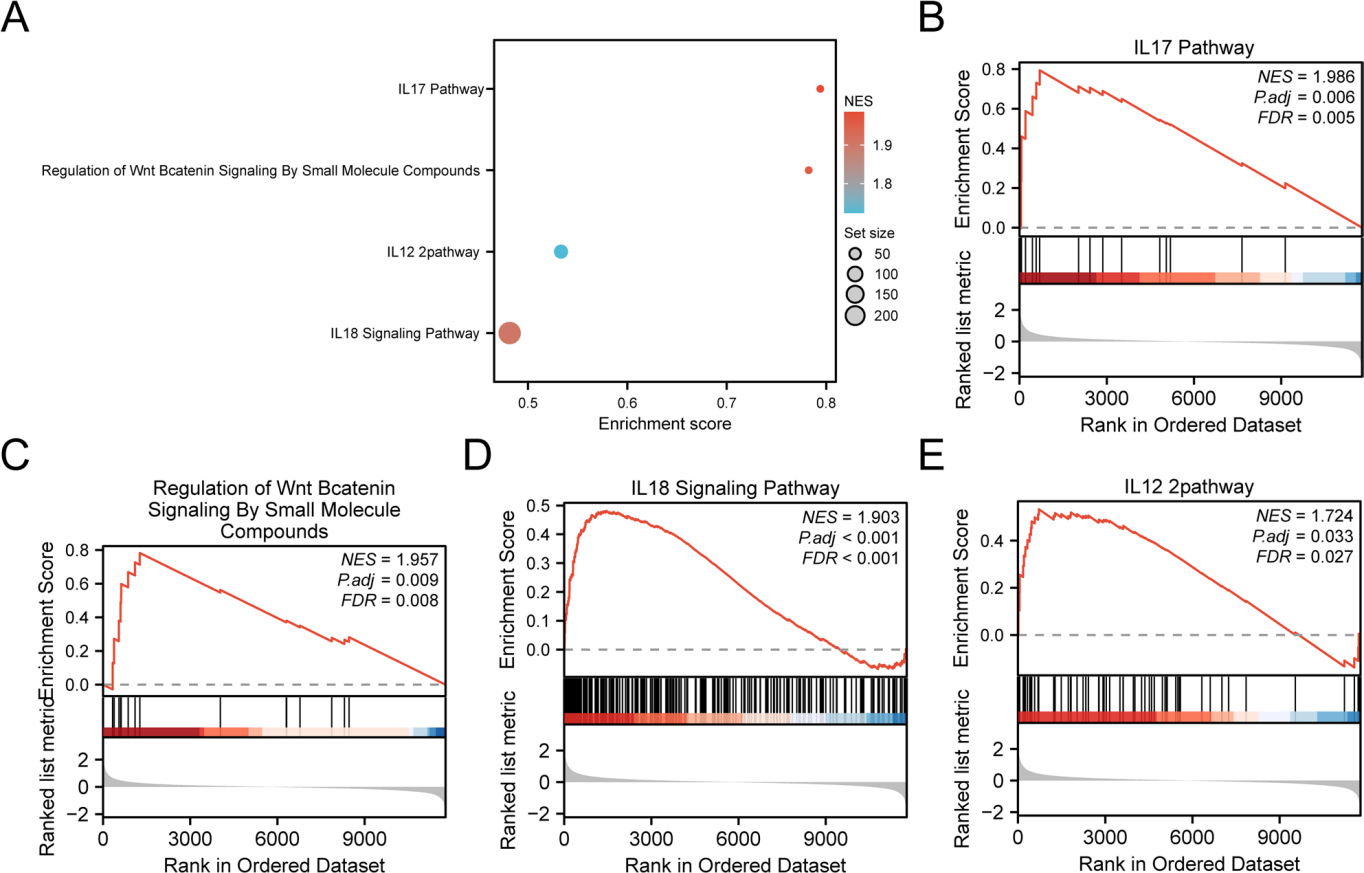
GSEA for combined datasets. **A**. Bubble plot presentation of four biological functions from GSEA of combined GEO datasets. **B–E**. GSEA showed that DKD was significantly enriched in the IL-17 signaling pathway (**B**), regulation of Wnt β-catenin signaling by small molecule compounds (**C**), and Wnt β-catenin signaling by small molecule compounds (**C**). IL-18 signaling pathway (**D**) and IL-12 signaling pathway (**E**). GEO, Gene Expression Omnibus; DKD, diabetic kidney disease; GSEA, gene set enrichment analysis

GSEA was performed to evaluate gene expression patterns and associated biological processes, cellular components, and molecular functions in the combined datasets (Fig. 5A; Table 3). Significant enrichment was observed in the IL-17 signaling pathway (Fig. 5B), regulation of Wnt/β-catenin signaling by small molecules (Fig. 5C), IL-18 signaling pathway (Fig. 5D), and IL-12 signaling pathway (Fig. 5E).

**Table 3:** Results of GSEA for combined datasets

ID	Set Size	Enrichment Score	NES	*p* value	*p*.adjust	q value
WP_TYROBP_CAUSAL_NETWORK_IN_MICROGLIA	50	8.20E-01	2.59E + 00	1.00E-10	3.43E-08	2.82E-08
WP_EXTRAFOLLICULAR_B_CELL_ACTIVATION_BY_SARSCOV2	65	7.69E-01	2.53E + 00	1.00E-10	3.43E-08	2.82E-08
WP_ALLOGRAFT_REJECTION	81	7.36E-01	2.51E + 00	1.00E-10	3.43E-08	2.82E-08
KEGG_SYSTEMIC_LUPUS_ERYTHEMATOSUS	49	7.93E-01	2.50E + 00	1.00E-10	3.43E-08	2.82E-08
REACTOME_IMMUNOREGULATORY_INTERACTIONS_BETWEEN_A_LYMPHOID_AND_A_NON_LYMPHOID_CELL	106	6.96E-01	2.47E + 00	1.00E-10	3.43E-08	2.82E-08
KEGG_LEISHMANIA_INFECTION	67	7.26E-01	2.39E + 00	1.97E-10	4.73E-08	3.89E-08
KEGG_ALLOGRAFT_REJECTION	33	8.28E-01	2.38E + 00	3.39E-09	4.97E-07	4.08E-07
REACTOME_INTEGRIN_CELL_SURFACE_INTERACTIONS	78	6.82E-01	2.31E + 00	2.99E-09	4.92E-07	4.04E-07
KEGG_CELL_ADHESION_MOLECULES_CAMS	115	6.14E-01	2.20E + 00	3.52E-09	4.97E-07	4.08E-07
REACTOME_INTERFERON_SIGNALING	176	5.74E-01	2.17E + 00	1.97E-10	4.73E-08	3.89E-08
REACTOME_NEUTROPHIL_DEGRANULATION	400	5.19E-01	2.15E + 00	1.00E-10	3.43E-08	2.82E-08
WP_NETWORK_MAP_OF_SARSCOV2_SIGNALING_PATHWAY	199	5.38E-01	2.06E + 00	2.27E-09	4.53E-07	3.73E-07
REACTOME_CLASS_A_1_RHODOPSIN_LIKE_RECEPTORS	265	5.20E-01	2.06E + 00	1.00E-10	3.43E-08	2.82E-08
REACTOME_EXTRACELLULAR_MATRIX_ORGANIZATION	259	5.13E-01	2.03E + 00	2.27E-10	4.95E-08	4.07E-08
KEGG_CYTOKINE_CYTOKINE_RECEPTOR_INTERACTION	230	5.17E-01	2.02E + 00	2.59E-09	4.78E-07	3.93E-07
BIOCARTA_IL17_PATHWAY	15	7.94E-01	1.99E + 00	3.13E-04	6.31E-03	5.19E-03
WP_REGULATION_OF_WNT_BCATENIN_SIGNALING_BY_SMALL_MOLECULE_COMPOUNDS	15	7.82E-01	1.96E + 00	5.39E-04	9.29E-03	7.64E-03
REACTOME_SIGNALING_BY_INTERLEUKINS	409	4.60E-01	1.91E + 00	1.23E-10	3.67E-08	3.02E-08
WP_IL18_SIGNALING_PATHWAY	244	4.82E-01	1.90E + 00	6.38E-08	4.86E-06	4.00E-06
PID_IL12_2PATHWAY	58	5.33E-01	1.72E + 00	2.60E-03	3.29E-02	2.70E-02

*GSEA* Gene Set Enrichment Analysis

### PPI network and hub genes

**Fig. 6:**
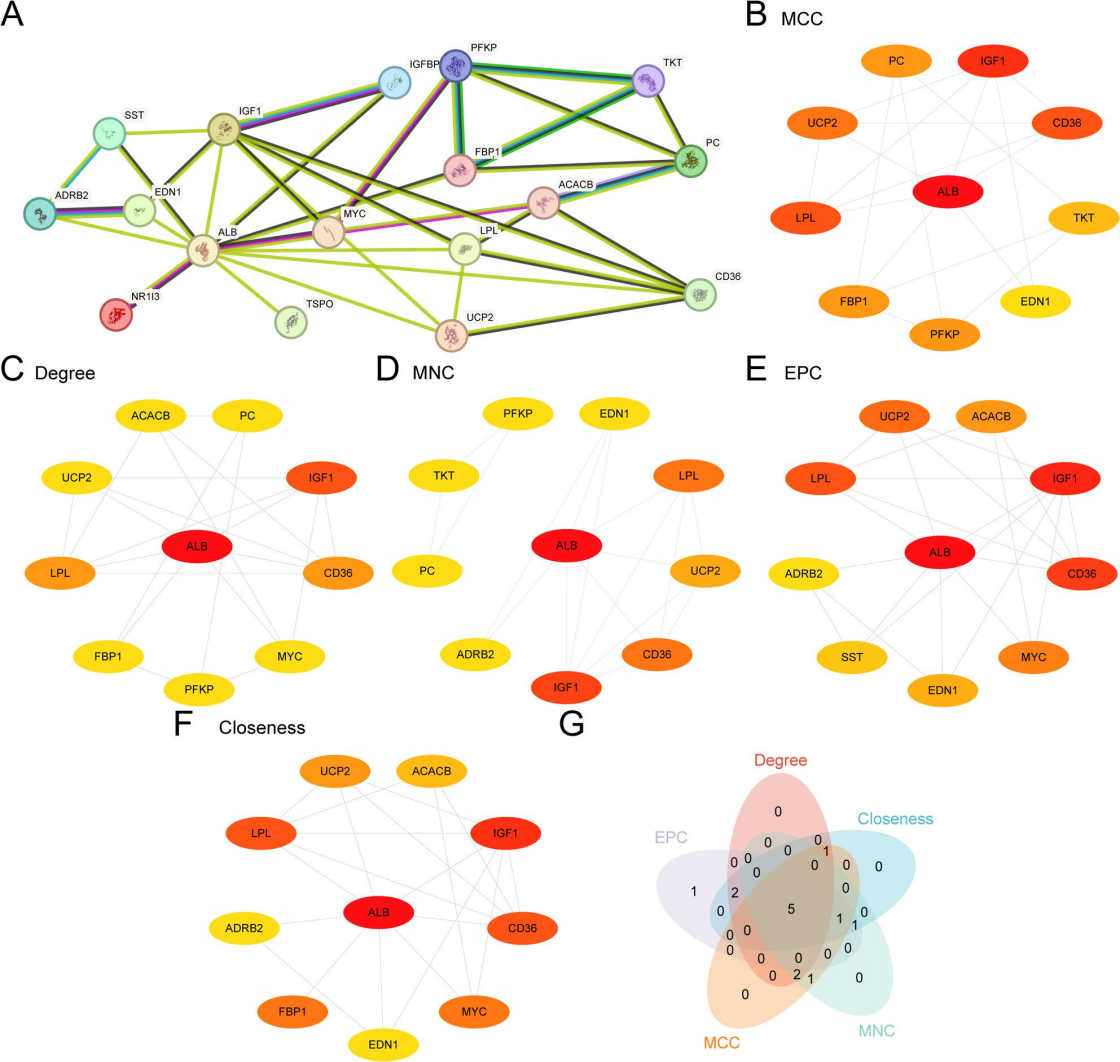
PPI network and hub genes analysis. **A**. PPI network of EMRDEGs calculated from the STRING database. **B–F**. PPI network of the top 10 EMRDEGs calculated using five algorithms of the cytoHubba plug-in, including MCC (Fig. 6B), Degree (Fig. 6C), MNC (Fig. 6D), EPC (Fig. 6E), and Closeness (Fig. 6F). Among them, the color of the circles, from red to yellow, represents the score from high to low. **G**. Venn diagram of the top 10 EMRDEGs for the five algorithms of the cytoHubba plug-in. EMRDEGs, energy metabolism-related differentially expressed genes; PPI, protein–protein interaction; MCC, maximal clique centrality; MNC, maximum neighborhood component; EPC, edge percolated component

A protein–protein interaction (PPI) network of 17 EMRDEGs (*UCP2*, *IGF1*, *TKT*, *CD36*, *ADRB2*, *TSPO*, *ACACB*, *LPL*, *FBP1*, *SST*, *IGFBP1*, *PFKP*, *NR1I3*, *MYC*, *ALB*, *PC*, and *EDN1*) was constructed using the STRING database (Fig. 6A). EMRDEGs were ranked by five topological algorithms (MCC, Degree, MNC, EPC, and Closeness), and the top 10 genes from each algorithm were visualized (Figs. 6B–F). The intersection of these top lists, demonstrated in a Venn diagram (Fig. 6G), yielded five hub genes: *ALB*, *IGF1*, *CD36*, *LPL*, and *UCP2*.

### Regulatory networks

**Fig. 7:**
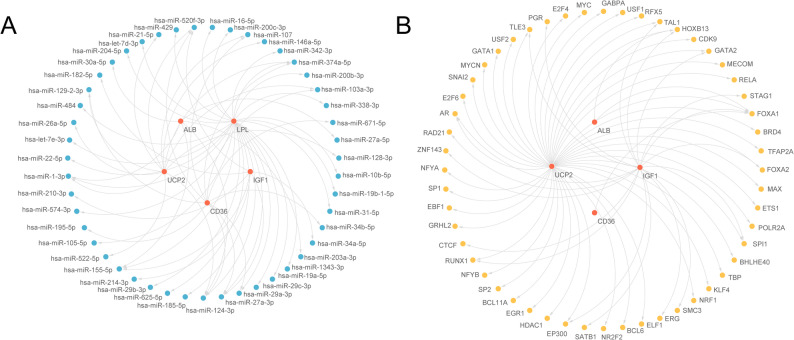
Regulatory network of hub genes. **A**. mRNA-miRNA Regulatory Network of hub genes. **B**. mRNA-TF Regulatory Network of hub genes. Red, blue, and yellow circles are mRNAs, miRNAs, and TFs, respectively. TFs, transcription factors

An mRNA–miRNA regulatory network comprising 47 miRNAs and the five hub genes was constructed in Cytoscape (Fig. 7A; Table S2).

TFs binding to hub genes were identified using ChIPBase and hTFTarget, and the intersecting set was rendered as an mRNA–TF regulatory network (Fig. 7B), which included 52 TFs and 4 hub genes (*ALB*, *IGF1*, *CD36*, *LPL*) (Table S3). Regulatory network analysis belongs to exploratory analysis and still requires experimental validation in subsequent steps to reflect the reasonable positioning of the research.

### Differential expression and ROC analysis of hub genes

**Fig. 8:**
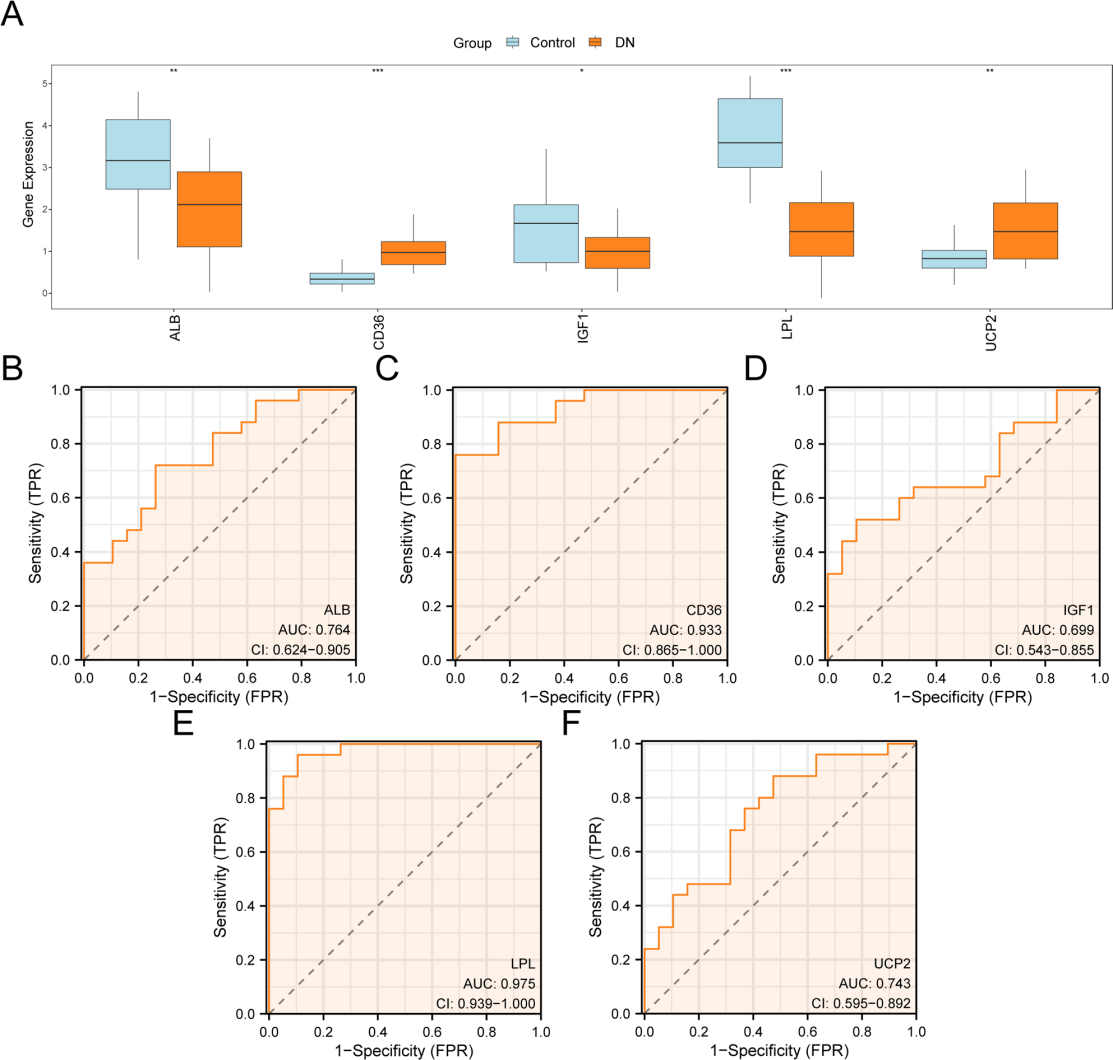
Expression difference and ROC curve analysis. **A**. Group comparison diagram of hub genes in combined GEO datasets. **B–F**: ROC curves of hub genes *ALB* (**B**), *CD36* (**C**), *IGF1* (**D**), *LPL* (**E**), and *UCP2* (**F**) with significant differences in expression values in the group comparison plot. * refers to *p* < 0.05, which is statistically significant; ** denotes *p* < 0.01, highly statistically significant; and *** means *p* < 0.001, extremely statistically significant. The closer the AUC was to 1, the better the diagnostic effect. The orange and blue represent the DKD and control groups, respectively. GEO, Gene Expression Omnibus; DKD, diabetic kidney disease; ROC, receiver operating characteristic; AUC, area under the curve

The Wilcoxon rank-sum test showed that expression levels of *CD36* and *LPL* were significantly higher in the DKD group than in the control group (*p* < 0.001; Fig. 8A). Significant differences were also observed for *ALB* and *UCP2* (*p* < 0.01) and for *IGF1* (*p* < 0.05) between the DKD and control groups.

ROC curve analysis (Fig. 8B) demonstrated that *CD36* (AUC = 0.933; Fig. 8C) and *LPL* (AUC = 0.975; Fig. 8E) had high diagnostic accuracy for DKD (AUC > 0.9). *ALB* (AUC = 0.764; Fig. 8B) and *UCP2* (AUC = 0.743; Fig. 8F) demonstrated moderate diagnostic accuracy (0.7 < AUC < 0.9), whereas *IGF1* (AUC = 0.699; Fig. 8D) demonstrated low diagnostic accuracy (0.5 < AUC < 0.7).

### Immune infiltration analysis of the DKD dataset

**Fig. 9:**
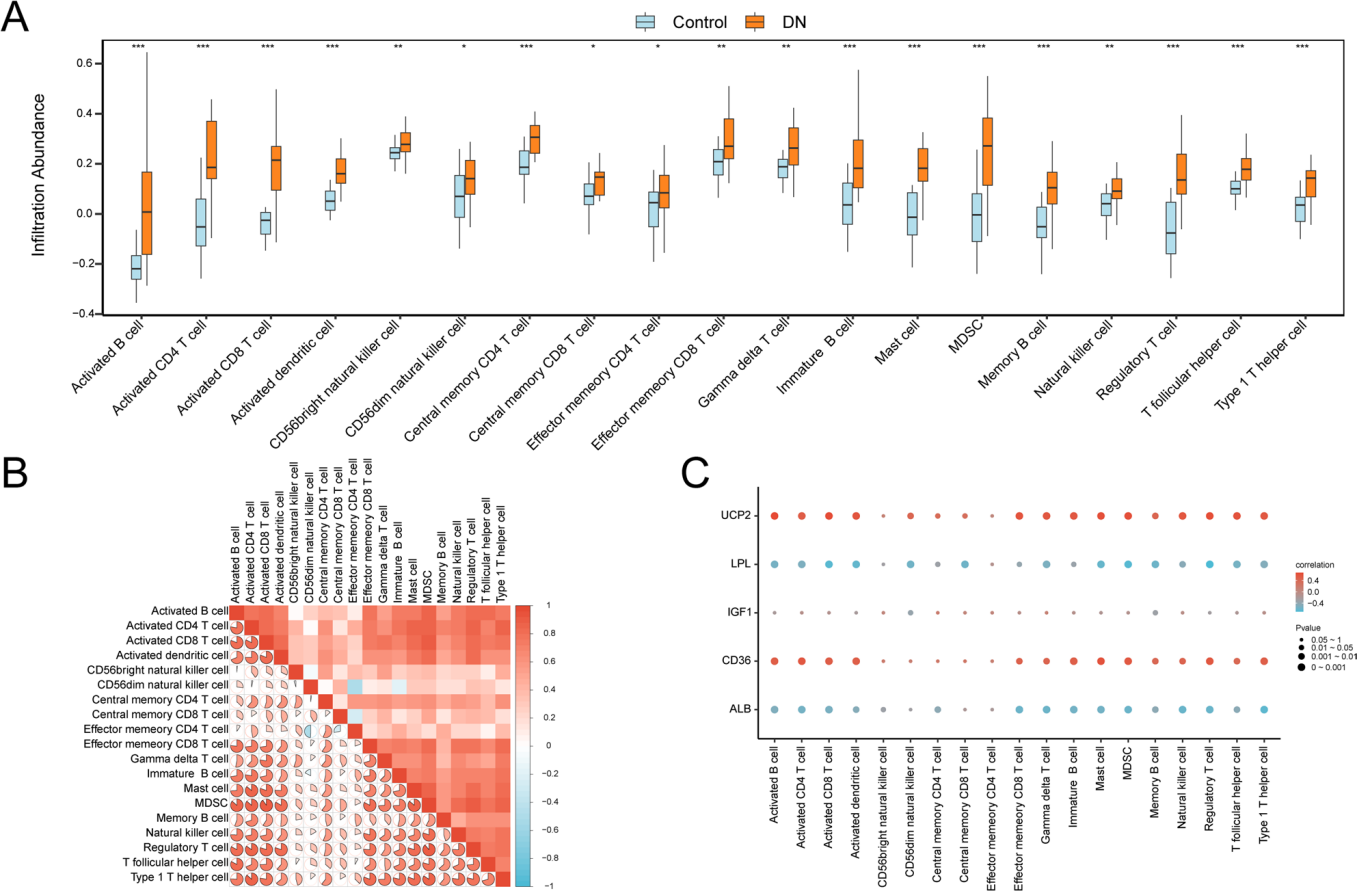
Combined datasets immune infiltration analysis using ssGSEA algorithm.** A **Plot of grouping comparisons of immune cells between the DKD and Control groups.** B **Heat map of the correlation between the infiltration abundance of immune cells with significant differences in infiltration abundance in the combined GEO datasets in group comparison plots.** C **Heat map of the correlation between hub genes and the infiltrating abundance of seven immune cells in integrated GEO datasets. * conveys *p* < 0.05, statistically significant; ** describes *p* < 0.01, highly statistically significant; and *** expresses *p* < 0.001, extremely statistically significant. It showed little or no correlation when the correlation coefficient (r value) was < 0.3, weak correlation when it was 0.3–0.5, moderate correlation when it was 0.5–0.8, and strong correlation when it was > 0.8. The DKD (orange) and control (blue) groups. GEO, Gene Expression Omnibus; DKD, diabetic kidney disease; ssGSEA, single-sample gene set enrichment analysis

Group comparisons (Fig. 9A) identified significant differences in 19 immune cell types between DKD and control samples (*p* < 0.05). The most pronounced differences (*p* < 0.001) were observed for activated B cells, activated CD4^+^ T cells, activated CD8^+^ T cells, activated dendritic cells, central memory CD4^+^ T cells, immature B cells, mast cells, myeloid-derived suppressor cells (MDSCs), memory B cells, regulatory T cells, T follicular helper cells, and type 1 T helper cells. Additional differences were detected for CD56bright natural killer (NK) cells, effector CD8^+^ T cells, and γδ T cells (*p* < 0.01), as well as for CD56dim NK cells and central memory CD8^+^ T cells (*p* < 0.05). Effector memory CD4^+^ T cells also differed significantly between groups (*p* < 0.05).

Correlation heatmaps (Figs. 9B–C) demonstrated strong positive correlations between activated CD8^+^ T cells and MDSCs (*r* > 0.8). Moderate positive correlations (0.5 < *r* < 0.8) were observed among central memory CD4^+^ T cells, activated CD4^+^ T cells, regulatory T cells, and T follicular helper cells. Weak positive correlations (0.3 < *r* < 0.5) were noted between effector memory CD4^+^ T cells and CD56bright NK cells, and between central memory CD4^+^ T cells and immature B cells, memory B cells, and γδ T cells. A weak negative correlation (− 0.5 < *r* < − 0.3) was observed between effector memory CD4^+^ T cells and CD56dim NK cells. Finally, correlations with hub genes showed that *UCP2* and *CD36* were positively associated with immune cell infiltration, while *LPL* and *ALB* were negatively associated with most immune cell types (Fig. 9C).

### IHC verification and clinical correlation analysis of key genes

**Fig. 10:**
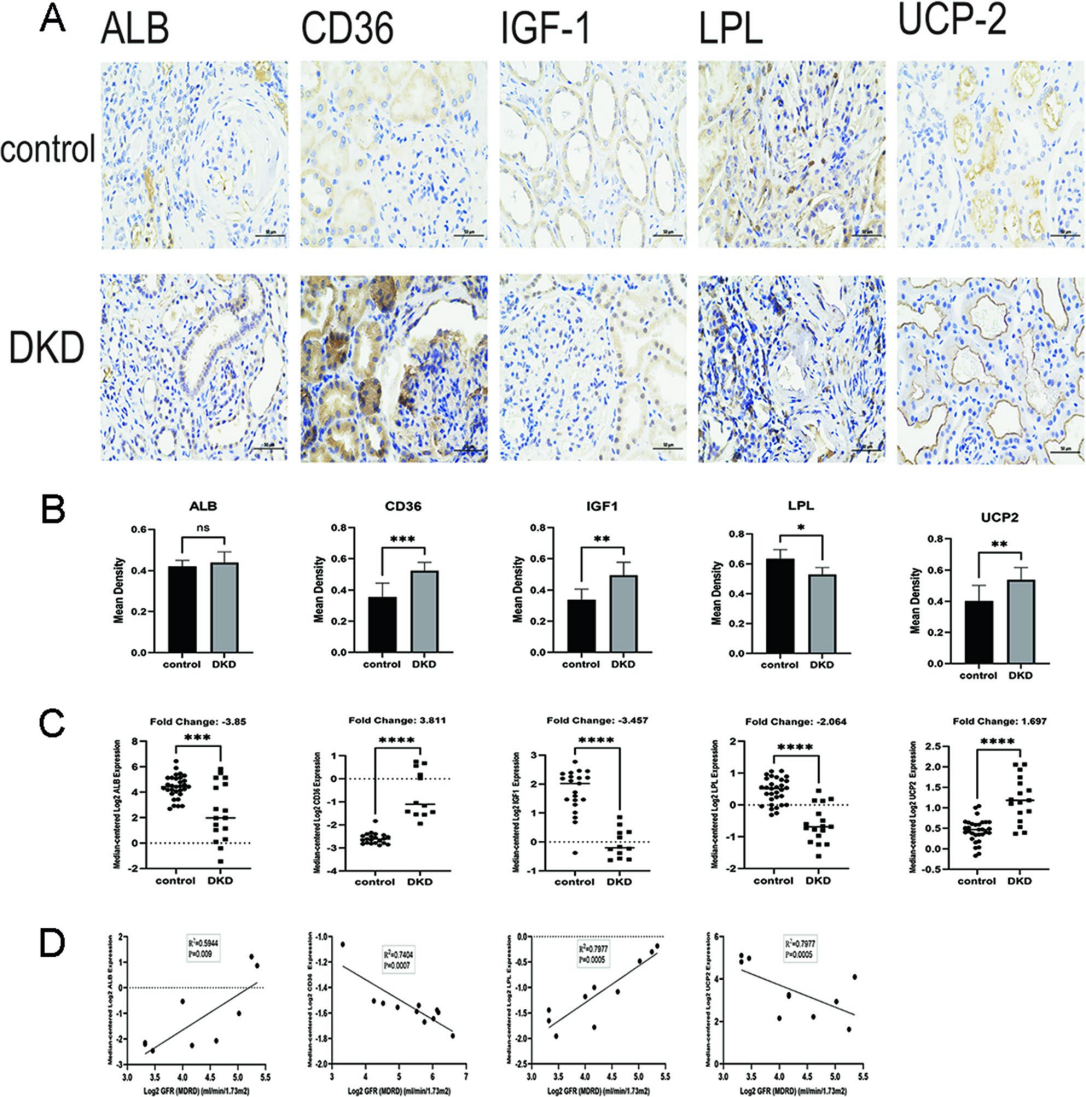
Immunohistochemical verification and clinical correlation analysis of key genes. **A** IHC staining was used to examine the expression of *ALB*,* CD36*,* IGF1*,* LPL*, and *UCP2* in six paracarinoma (con) and 10 DKD kidney tissues (scale bar, 50 μm; magnification, 400×). **B** IHC quantitative expression statistics (mean s.d.). **C** Identification of *ALB*,* CD36*,* IGF1*, *LPL*, and *UCP2* expression in DKD with healthy individuals as control using Nephroseq database. **D** Correlation analysis of *ALB*,* CD36*,* LPL*, and *UCP2* expression with GFR. ns, no significance, *p < 0.05, **p < 0.01, ***p < 0.001, and ****p ≤ 0.0001

Immunohistochemistry (IHC) of kidney biopsies showed significant differences in protein expression of *CD36*, *IGF1*, *LPL*, and *UCP2* between DKD and control groups (Figs. 10A–B).

Analysis in NephroSeq revealed that *CD36* was upregulated (*p* < 0.0001; fold change = 3.811), *IGF1* and *LPL* were downregulated (*p* < 0.0001; fold changes = − 3.457 and − 2.064, respectively), and *UCP2* was upregulated (*p* < 0.0001; fold change = 1.697) in DKD (Fig. 10C).

Correlation analysis with GFR indicated strong positive associations for *ALB* and *LPL*, and significant negative associations for *CD36* and *UCP2* (Fig. 10D).

## Discussion

DKD is the primary cause of end-stage renal disease, accounting for 40% to 50% of individuals requiring dialysis, and substantially contributes to both mortality and morbidity [27, 28]. Although albuminuria and estimated GFR are the most frequently used indicators of DKD, their limitations have driven the search for more reliable biomarkers for earlier detection and targeted therapeutic interventions. Recent studies investigating altered energy metabolism in DKD have provided important insights. Hyperglycemia can increase the production of reactive oxygen species (ROS) and induces oxidative stress, thereby exacerbating renal injury [29, 30]. Nevertheless, additional molecular targets and therapeutic strategies remain to be identified.

In this study, five hub genes associated with energy metabolism in DKD were identified: *CD36*,* LPL*,* ALB*,* UCP2*, and *IGF1*. ROC curve analyses demonstrated that *CD36* and *LPL* had high diagnostic accuracy, *ALB* and *UCP2* demonstrated moderate accuracy, and *IGF1* had low diagnostic accuracy. Furthermore, GO, KEGG, and GSEA analyses indicated that these EMRDEGs were significantly enriched in multiple energy metabolic pathways. IHC analysis of renal biopsy specimens revealed statistically significant differences in *CD36*, *IGF1*, LPL, and *UCP2* expression between DKD and control groups. Collectively, these results suggest that *CD36*, *IGF1*, *LPL*, and *UCP2* play important roles in linking DKD to altered energy metabolism.

ROC curve analysis further reinforced the diagnostic potential of the hub genes, particularly *CD36* and *LPL*, which had AUC values exceeding 0.9, indicating strong biomarker potential for DKD detection. Expression analysis from IHC data and the Nephroseq V5 database confirmed that *CD36* was overexpressed and LPL was downregulated in DKD. *CD36*, a transmembrane glycoprotein, mediates the transport of long-chain fatty acids across membranes and is implicated in metabolic inflammation [31]. Hyperglycemia and lipid abnormalities, characterized by increased intracellular and plasma fatty acid concentrations, are closely linked to DKD. In high-glucose (HG)-treated HK-2 cells, *CD36* expression was elevated, facilitating the uptake and intracellular trafficking of free fatty acids [32]. Evidence indicates that oxidative stress resulting from lipid accumulation is a major mechanism by which *CD36* contributes to DKD pathogenesis [33]. Findings of elevated *CD36* expression in renal tissue from patients with DKD are consistent with the results [34]. 

Prior studies have reported that metformin may counteract oxidative stress–induced increases in *CD36* levels in pancreatic β cells [35]. Collectively, these observations support *CD36* as both a promising biomarker and a potential therapeutic target for DKD. Previous research has demonstrated that DKD is associated with markedly reduced renal *LPL* mRNA expression, accompanied by elevated triglyceride (TG) levels in kidney tissue [36]. Angiopoietin-like 4 (Angptl4) has been identified as a potent inhibitor of *LPL*, regulating TG uptake by cells and enhancing fatty acid oxidation [37]. 

The development of microvascular complications, including DKD, and cardiovascular disorders associated with diabetes is strongly influenced by oxidative stress [38]. Mitochondrial ROS have been identified as key mediators of hyperglycemia-induced tissue injury [39]. Therefore, strategies targeting mitochondrial ROS production may help mitigate oxidative stress and slow DKD progression.

*UCP2*, a member of the mitochondrial carrier protein family, reduces adenosine triphosphate synthesis, lowers the electrochemical gradient across the mitochondrial membrane, and decreases ROS generation [40]. In this study, *UCP2* expression differed significantly between the DKD and control groups (*p* < 0.01) and demonstrated moderate diagnostic accuracy. IHC results from renal biopsy specimens and Nephroseq V5 data confirmed *UCP2* overexpression in DKD. The results of previous studies indicate that elevated intracellular ROS levels can upregulate *UCP2* mRNA expression, and that overexpression of *UCP2* analogs or antioxidant enzymes may reduce ROS levels, thereby preventing diabetes-related complications [41, 42]. These findings highlight *UCP2* as a promising therapeutic target for DKD.

Previous studies have also indicated an association between circulating *IGF1* levels and DKD [43]. Some reports indicate that individuals with diabetic nephropathy exhibit elevated serum *IGF1* levels that increase with disease progression [44]. In contrast, the bioinformatics analysis and Nephroseq V5 results showed that *IGF1* expression was downregulated in DKD, contradicting both the IHC results and earlier studies. The causes of these discrepancies remain unclear, underscoring the need for further investigation into the underlying mechanisms.

GO and KEGG pathway enrichment analyses revealed that EMRDEGs were predominantly enriched in energy metabolism–related pathways, particularly the AMPK signaling pathway. In multicellular organisms, AMPK coordinates energy metabolism at the systemic level [45]. AMPK also functions as a physiological regulator of ROS, protecting endothelial cells from hyperglycemia-induced damage by suppressing oxidant production [46]. Given its central role in energy homeostasis, the AMPK signaling pathway represents a potential therapeutic target for metabolic disorders, including DKD. For instance, colquhounia root tablets have been shown to exert protective effects in vivo and in vitro by reducing *CD36* expression and activating AMPK, thereby promoting autophagy and inhibiting apoptosis [47]. 

GSEA showed significant enrichment of genes in the IL-17 signaling pathway across the combined GEO datasets. Elevated IL-17 levels have been reported in several metabolic diseases, including diabetes [48]. In diabetic models, IL-17-deficient mice exhibited reduced glomerular damage, decreased fibrosis, and lower albuminuria, indicating that IL-17 signaling contributes to DKD pathogenesis [49]. Moreover, early-stage DKD may depend on IL-17 pathway activation, and administration of low-dose recombinant IL-17 A has been suggested as a potential preventive and therapeutic strategy [50, 51]. 

ssGSEA–based immune infiltration analysis revealed significant differences in 19 immune cell types, including mast cells, activated CD4^+^ T cells, and activated CD8^+^ T cells. Previous studies have shown that individuals with diabetes have elevated CD8^+^ T-cell counts, and that inhibiting these cells can attenuate DKD-associated pathology [52]. Pro-inflammatory cytokines released by activated CD4^+^ and CD8^+^ T cells can stimulate macrophages directly or indirectly via mesangial cell production of monocyte chemoattractant protein-1 and colony-stimulating factor-1 [53]. Activated macrophages, in turn, release mediators such as nitric oxide, ROS, IL-1, tumor necrosis factor alpha (TNF-α), complement components, and metalloproteinases, all of which contribute to renal injury [54, 55]. The extent of T-cell infiltration into renal tissue correlates with albuminuria severity, and abatacept has been shown to reduce DKD progression by preventing systemic T-cell activation [56]. Similarly, mast cell counts increase with DKD progression, and their activation is strongly associated with tubulointerstitial damage [57, 58]. Preclinical studies have demonstrated that mast cell inhibitors, such as cromolyn and ketotifen (Zaditor), protect diabetic mice from kidney injury [59]. 

This study utilizes integrated bioinformatics and in vivo analysis to identify key genes associated with energy metabolism in diabetic nephropathy. The next step involves constructing cellular models and animal models of diabetic nephropathy to investigate the expression of key genes and their associated pathways and molecules, thereby clarifying how these genes contribute to the onset and progression of diabetic nephropathy. These findings will facilitate the discovery of novel diagnostic markers and therapeutic targets for diabetic kidney disease (DKD), paving the way for innovative treatment strategies.

This study has several limitations. The analysis is primarily based on publicly available datasets from the GEO database. Although batch effect correction was performed using the R package sva and standardized processing was applied to minimize technical biases, potential clinical heterogeneity still exists among samples from public databases. Differences in age, duration of diabetes, stages of kidney disease progression, as well as variations in previous or concurrent treatment regimens among different subjects may all exert certain influences on gene expression profiles and subsequent analysis results.

## Conclusion

In conclusion, *CD36*,* IGF1*,* LPL*, and *UCP2* appear to be key EMRGs in DKD and may serve as novel diagnostic and therapeutic targets. These findings advance understanding of the metabolic underpinnings of DKD and provide a foundation for future studies to clarify the precise biological roles of these genes in disease progression.

## Supplementary Information

Supplementary Material 1.

Supplementary Material 2.

Supplementary Material 3.

## Data Availability

All data generated or analysed during this study are included in this article. Further enquiries can be directed to the corresponding author.

## Abbreviations

ALB: Albumin
AUC: Area under the curve
BP: Biological processes
CC: Cell component
CD28: Cluster of differentiation 28
CD36: Cluster of differentiation 36
CTLA-4: Cytotoxic T-lymphocyte–associated protein 4
DCs: Dendritic cells
DEGs: Differentially expressed genes
DKD: Diabetic kidney disease
EDN1: Endothelin 1
EPC: Edge percolated component
EMRGs: Energy metabolism-related genes
EMRDEGs: Energy metabolism-related differentially expressed genes
FBP1: Fructose-bisphosphatase 1
FDR: False discovery rate
GEO: Gene Expression Omnibus
GFR: Glomerular filtration rate
GO: Gene Ontology
GSEA: Gene set enrichment analysis
HG: High glucose
HK-2: Human kidney-2 cell line
IHC: Immunohistochemistry
IGF1: Insulin-like growth factor 1
IGFBP1: Insulin-like growth factor binding protein 1 IL interleukin
KEGG: Kyoto Encyclopedia of Genes and Genomes
LPL: Lipoprotein lipase
MCC: Maximal clique centrality
MDSC: Myeloid-derived suppressor cell
MF: Molecular function
miRNA: microRNA
MNC: Maximum neighborhood component
mRNA: messenger RNA
MSigDB: Molecular Signatures Database
NO: Nitric oxide
PCA: Principal component analysis
PFKP: Phosphofructokinase, platelet
PPI: Protein–protein interaction
ROC: Receiver operating characteristic
ROS: Reactive oxygen species
ssGSEA: Single-sample gene set enrichment analysis
SST: Somatostatin
STZ: Streptozotocin
TF: Transcription factor
TFs: Transcription factors
TNF-α: Tumor necrosis factor alpha
TSPO: Translocator protein
UCP2: Uncoupling protein 2
